# A Theoretical Framework of Haptic Processing in Automotive User Interfaces and Its Implications on Design and Engineering

**DOI:** 10.3389/fpsyg.2019.01470

**Published:** 2019-07-26

**Authors:** Stefan Josef Breitschaft, Stella Clarke, Claus-Christian Carbon

**Affiliations:** ^1^BMW Group, Munich, Germany; ^2^Bamberg Graduate School of Affective and Cognitive Sciences (BaGrACS), Bamberg, Germany; ^3^Department of General Psychology and Methodology, University of Bamberg, Bamberg, Germany

**Keywords:** haptics, automotive, user experience, framework of haptic processing, interaction design, haptic design, haptic interface

## Abstract

Driving a car is a highly visual task. Despite the trend towards increased driver assistance and autonomous vehicles, drivers still need to interact with the car for both driving and non-driving relevant tasks, at times simultaneously. The often-resulting high cognitive load is a safety issue, which can be addressed by providing the driver with alternative feedback modalities, such as haptics. Recent trends in the automotive industry are moving towards the seamless integration of control elements through touch-sensitive surfaces. Psychological knowledge on optimally utilizing haptic technologies remains limited. The literature on automotive haptic feedback consists mainly of singular findings without putting them into a broader user context with respect to haptic design of interfaces. Moreover, haptic feedback has primarily been limited to the confirmation of control actions rather than the searching or finding of control elements, the latter of which becomes particularly important considering the current trends. This paper presents an integrated framework on haptic processing in automotive user interfaces and provides guidelines for haptic design of user interfaces in car interiors.

## Introduction

**Figure 1:**
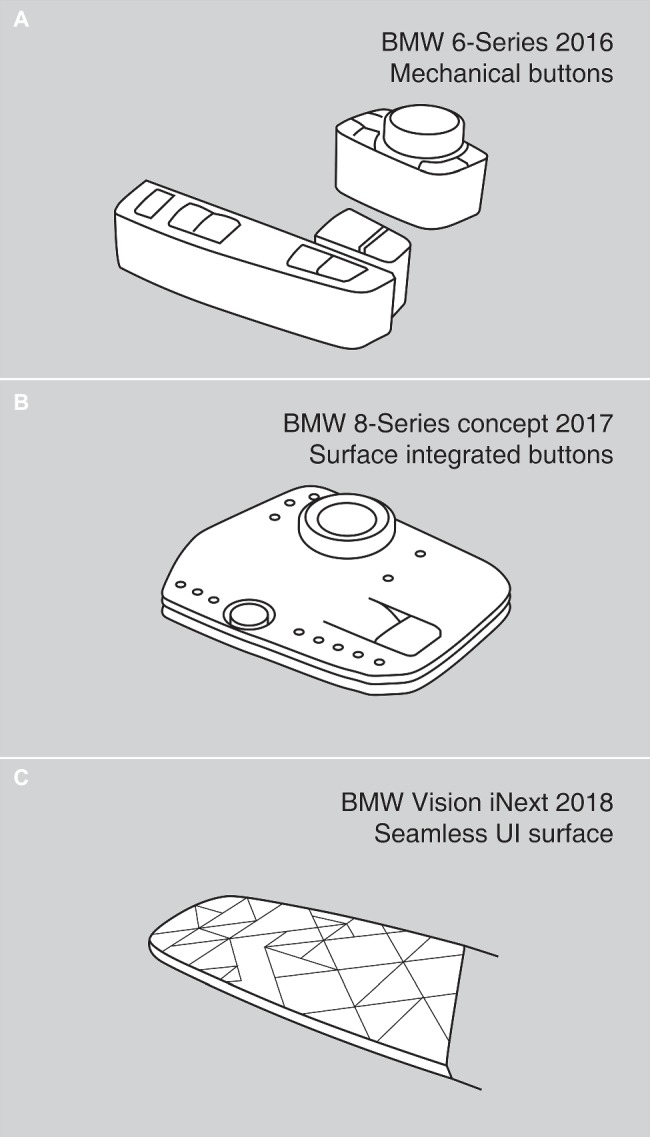
Exemplified depiction of the shift from **(A)** mechanical buttons (BMW Group, 2015) through **(B)** surface-integrated buttons (BMW Group, 2017) to **(C)** seamlessly integrated UI-surfaces (BMW Group, 2018).

Studies on haptic feedback in automotive use cases usually start with several remarks about the staggering increase in control options in car interiors and how they are overwhelming for users in terms of cognitive load and distraction in driving situations. Deploying haptic perception reduces reaction time and alleviates cognitive load in the visually demanding task of driving; ultimately, it increases safety (Petermeijer et al., 2015). However, most studies concentrate on technology-based haptic innovations, incorporating aspects such as proof of concept, usability tests, and a strong focus on technical solutions (Banter, 2010; Gaffary and Lécuyer, 2018). This one-eyed landscape of research literature makes it difficult for most stakeholders (such as designers, engineers, and usability researchers) to understand and predict why certain types of haptic feedback in user interfaces succeed while others fail. This is especially true for the automotive industry in which user interfaces need to be intuitive and usable. Practitioners in the automotive industry are currently facing disruptive changes. Through changing perspectives on mobility concepts, functionality in car interiors is dramatically increasing, positively influencing the demand for more flexible, adaptive and intelligent interface solutions. Design studies by different car manufacturers and automotive suppliers depict a clear vision of future car interiors and user interfaces. Concept studies, such as the BMW Vision iNEXT (BMW Group, 2018), are dominated by clean and harmonic interior surfaces that incorporate not only multiple layers of functionality (e.g., sensing, lighting, and haptics) but also a wide variety of new materials (metal, wood, and textiles) in user interfaces (Aito, 2018; Preh, 2018; QUAD Industries, 2018). These so-called “smart surfaces” facilitate the development of dynamically responding and context-sensitive surfaces in interaction situations. The appearance of user interfaces in the car has changed quite drastically in recent years. The amount of visible buttons in car interiors decreases through the seamless integration of interaction panels into design surfaces (see Figure 1) and the use of touchscreens. Active haptic technologies support the trend toward surface integration by enabling tactile feedback on seamless surfaces (Lust and Schaare, 2016; Aito, 2018). Most touchscreens and touch-sensitive surfaces, though being potentially aesthetically pleasing, nowadays lack specific haptic feedback. As a consequence, drivers wishing to find elements on a control panel may need to take their eyes off the street, creating potential safety concerns. This is especially true for the use of large touchscreen interfaces, such as in the current Tesla Model S. Budiu (2019) concludes that virtual buttons require a lot of visual attention without the use of haptic feedback. Using haptic feedback as a technological enabler for surface or optimized search haptics is not yet widespread. Although novel and technically promising solutions exist, many of these lack psychological data and knowledge of human perception.

With new trends, such as autonomous driving, a drastic increase in touch-sensitive surfaces and the use of new materials in interfaces, psychology-based knowledge on haptic perception and user experience is becoming more important. However, literature on the use of haptic feedback in automotive user interfaces is somewhat fragmented and therefore hard to overlook for practitioners. What is essentially missing is a comprehensive theoretical framework on how users perceive haptic feedback in user interfaces while controlling certain functions.

### Motivation and Aim of the Present Paper

The major goal of the this paper is to establish a theoretical framework that outlines and explains haptic processing in automotive user interfaces based on findings from usability studies, perception science, and cognitive psychology. A well-designed haptic interface can help keep the user’s eye on the road while controlling, and thus increases driving safety and performance (Kern and Pfleging, 2013).

Current user-centered research on automotive haptic feedback struggles to provide sufficient answers for the challenges posed by the abovementioned trends. Also case studies on currently used interfaces, such as done by Budiu (2019), claim the necessity of haptic feedback in automotive interfaces. Usability studies focus on comparing different interface concepts and technologies by using objective usability measures such as task completion time and error rates. User experience^1^ (UX)-factors giving insights on how users interpret certain aspects of the haptic feedback are often neglected. A multitude of studies resembles pure feasibility tests of novel haptic technologies. Generalizability of findings is somewhat limited due to the characteristics of certain haptic technologies. Studies on haptic feedback mostly focus on confirmation and pressing haptics, which overlooks other important steps in the interaction process. A majority of studies reports the effectiveness of haptic feedback on driving safety, but does not provide guidelines as to how haptic feedback can be optimized (for review, see Gaffary and Lécuyer, 2018 and Petermeijer et al., 2015). Findings are hardly put into a broader user experience context, which leads to a lack of best practices and guidelines on haptic design of automotive user interfaces.

A theoretical framework of literature on haptic feedback (1) enables the detection of gaps in research regarding future automotive haptic technologies, (2) helps stakeholders cope with the challenges and chances posed by human haptic perception, and (3) gives engineers guiding principles in the haptic design of automotive user interfaces.

This paper is structured as follows: (1) a brief overview of the literature on haptic perception and feedback in automotive user interface contexts will be presented, (2) an introduction of a common terminology of haptic feedback in automotive user interfaces will be given, (3) a theoretical framework of haptic processing in automotive interiors will be presented, and (4) practical implications and paradigmatic applications of the framework will be discussed.

## Haptic Perception in Automotive Interiors

To understand the complexity of haptic stimuli in automotive interiors, this paper gives a short introduction into haptic perception with respect to its importance in automotive interfaces. For a more general introduction into haptic perception, see Lederman and Klatzky (2009). In the automotive context, the haptic modality is often referred to as a channel capable of alleviating visual and cognitive load (Gaffary and Lécuyer, 2018). This is due to the high amount of visual information that is assumed to be processed during a driving task (Vollrath and Krems, 2011, p. 29). Haptic information in automotive contexts mostly originate from both kinesthetic and tactile (or cutaneous) stimuli. Much of the driving relevant haptic information perceived during driving is not deliberately conveyed *via* user interfaces. These undeliberate information can range from acceleration or lateral forces conveyed by muscles and tendons to vibrations felt due to bumpy road conditions. Nevertheless, focus of user interface designers is to deliberately use haptic feedback for certain use cases. van Erp and van Veen (2001) describe five different categories of how haptic vibrotactile information can be used in vehicles: warning, spatial, communication, coded, and general information (see Table 1). The increasing use of different surface materials also means that haptic surface properties need to be taken into account as a deliberate application of haptic perception. There is an increasing body of research dealing with the aesthetical aspects of material properties (Etzi et al., 2014). Carbon and Jakesch (2013) stress in their “model for haptic aesthetic processing” that with higher cognitive processing of haptic objects, aesthetical and utilization evaluations are integrated into perception of an object. During exploration, associations such as pleasantness and arousal are connected with the explored surfaces and materials. An example is the high quality feel of heavy and sturdy objects. Therefore, the list of haptic information categories proposed in Table 1 may be extended by aestehtic impressions.

**Table 1:** Classes of haptic information in automotive interior based on van Erp and van Veen (2001).

Category	Description	Possible application
Spatial	Using haptic information to indicate the location of important objects	Awareness of surrounding,^1^ blind spot^2^
Warning	Using haptic information to warn the driver in dangerous situations	Lane departure,^1^ collision prevention^1^
Communication	Using haptic information as a subtle communication channel	Navigation^1^
Information	Using haptic information to display current status information regarding the car	Speed control,^1^ Maneuver support,^1^ eco-friendly^2^
Interaction	Using haptic information in interaction with control units	Controlling the car’s functions^1^
Aesthetical^3^	Using haptic information to evoke aesthetical appreciation^4^	Brand image,^3^ perceived quality^5^

*Possible applications are allocated to a single category for a clear overview. However, a few applications may overlap in their information category, such as lane departure, whose main goal is to warn the driver, but also gives information on the location on the road*.

1 *Gaffary and Lécuyer (2018)*,

2 *Petermeijer et al. (2015)*,

3 *Carbon and Jakesch (2013)*,

4 *Swindells et al. (2007)*,

5 *Glohr et al. (2015)*.

### Haptic Feedback in Automotive User Interfaces

This section gives a short overview on the literature of haptic feedback in situations of controlling a car’s function. Gaffary and Lécuyer (2018) and Petermeijer et al. (2015) give a broad overview on the use and effectiveness of haptic feedback in the automotive context. They emphasize the impact of haptic feedback on driving safety, reaction time, and driver performance. There are also studies challenging these findings, for instance, Pitts et al. (2012a) found a mixed influence of haptic feedback on driving relevant experimental variables. Performance in a lane change test did not significantly differ across different feedback modalities (visual, visual + haptic, visual + audio, visual + haptics + audio). Haptic feedback was chosen based on preference ratings in a preliminary study. However, in the main study, participants reported that haptic feedback in the visual + haptic, and also in the visual + audio + haptic condition was not strong enough to be perceived robustly. Participants showed a preference for combined visual and auditory feedback in confidence and hedonic ratings. Possibly, the choice of haptic feedback impulse was an influencing factor in this outcome. In fact, in an earlier study, Pitts et al. (2009) pointed out an increase in acceptance and user experience when haptic feedback is involved. Pitts et al. (2010) were able to show an increase in confidence with the haptic feedback modality. Also, Weddle and Yu (2013) found that users felt more confident and pleasant by using an interface with haptic feedback in comparison to a non-haptic tablet. Furthermore, Pitts et al. (2012a) found that the importance of haptic feedback increases when visual feedback is delayed in interactive situations. But also within the haptic modality, high latency from touch to feedback can already decrease task performance and satisfaction (Weddle et al., 2013). Rydström et al. (2009) concluded that the usage of haptic information in control units encourages users to keep their eyes on the road. Since haptic exploration is a serial perception process, subjects tend to take longer for task completion, and hence mainly rely on visual information. Nonetheless, Burnett and Irune (2009) describe haptic perception as a key indicator of perceived quality in car switches. Ng et al. (2017) compared direct touch, pressure-based-touch, and a physical dial as possible input methods and found that pressure-based touch took the longest and produced more but shorter glances than only touch input. They again found shorter task completion times and higher preference ratings for pressure-based buttons and the turning knob with haptic feedback in a previous study (Ng and Brewster, 2016). Richter et al. (2010) conducted a preliminary study with their *HapTouch*-Device, a force-sensitive input device with haptic feedback, which showed reduced error rates and task completion times for a haptic feedback condition. They reported a positive influence of haptic feedback on small and large touchscreen devices—although this finding is based on a small sample size of five participants only. Grane and Bengtsson (2012) found that haptic feedback in rotary control elements can produce fewer turn errors and lower task completion times than pure visual feedback. However, they could not detect a correlation between the amount of haptic information and errors or time. Kern and Pfleging (2013) concluded that haptic feedback can be helpful in many driving situations when the implementation of the feedback is carefully considered.

In summary, automotive user interfaces can benefit from the use of haptic feedback, despite challenging findings in multisensory feedback settings. An explanation for the mixed findings may be that the effectiveness of haptic feedback strongly depends on the specific use and implementation of the haptic feedback technology. Unfortunately, there are only few studies examining best practices on how to seamlessly integrate different haptic technologies into interaction concepts.

### Common Terminology on Haptic Feedback

**Figure 2:**
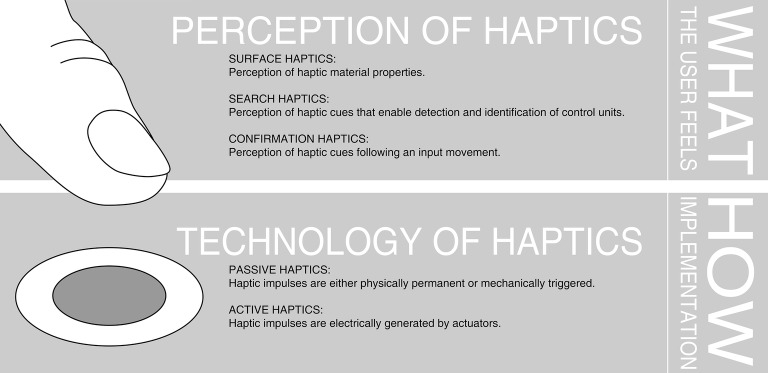
Overview on different terminologies of haptics in the context of automotive interiors. Haptics in automotive user interfaces contains a perception and technological part.

In recent years, there is a growing number of companies, especially in the consumer electronic industry, developing and delivering innovative haptic feedback solutions. Many of these tech companies use their own wording to describe their feedback technology. This makes it hard for OEM-developers and consumers to compare different technologies. Additionally, as the research community mainly refers to haptic feedback, there is also a lack of common terminology in the scientific area. Hence, the following section gives a categorization on different terminologies of haptics that are being used in the automotive context. Figure 2 shows various meanings of the term “haptics” in the context of car interiors. In general, haptics in control elements can be viewed from a technological (How is haptic feedback generated?) and a perceptual perspective (What can be felt by the user? How are the haptic impulses interpreted?). This separation can also be found in Bubb et al. (2015). We will follow a psychological view, where we differentiate between search and confirmation haptics. In the context of searching and identifying user interfaces, surface haptics can be referred to as search haptics (see Figure 2).

#### Surface and Search Haptics

*Surface haptics* refers to all haptic surface and material properties that can be used in car interiors. One of the rare examples of haptic processing models is the “model of haptic aesthetic processing” developed by Carbon and Jakesch (2013). They presume that high-level processing of haptic stimuli involves utilization and aesthetic evaluation. This means, interior materials convey hedonic as well as functional aspects. Regarding hedonic aspects, surface haptics refers to the use of haptics to evoke aesthetical evaluations. For example, certain surface materials are used to underline certain associations, such as perceived quality, coziness, or warmth, connected to the car (Bubb et al., 2015, p. 285). However, in automotive user interfaces surface properties such as joints, edges, recesses, other surface geometries, and textures can also have a clear functional reason, for instance, to support drivers in the blind operation of car interfaces to free up visual resources for the primary driving task.

*Search haptics* refers to the functional use of haptic surface properties, such as joints, ridges, edges, detents, surface geometries and textures as orientation, indication and separation cues to interactive areas in a car interior. Search haptics has so far mainly been a by-product of the mechanical integration of control elements, such as, gaps between buttons. There is little research on how to use haptic cues to optimally support users in finding interactive areas, differentiating between adjacent control elements, and giving users orientation in interior surfaces. The lack of research becomes even more obvious with an increasing use of seamless interactive surfaces in nowadays designs, such as touch and gesture-sensitive surfaces.

#### Confirmation Haptics

*Confirmation haptics* refers to clear and specific haptic feedback for changing the operation status of a control element. Confirmation cues can be manifold. The simplest example is the feel of a button click after pressing. The aim of confirmation haptics is to give users a clear and distinctive haptic feedback to increase the guidance for blind operation and ultimately, to increase perceived as well as objective safety.

Still, every interaction concept is based on a technological counterpart – the hardware. In the automotive industry, haptic technologies can be separated into two categories: passive and active haptic feedback technologies – or short *passive* and *active* *haptics*. Both of these can be applied to search and confirmation haptics.

#### Passive Haptics

*Passive haptics* refers to haptic feedback generated by mechanical elements, or physically anchored and permanent stimuli. It involves no external electrical energy input. The energy is generated by pressure from the user, and the haptic feedback is generated by the reaction of the mechanical elements to this energy.

Within surface and search haptics, passive haptics refers to non-changing surface shapes, geometries, and textures. On a computer keyboard, for instance, the hardly noticeable (and in fact widely unnoticed) bumps on the *F* and *J* keys are also a form of passive search haptics^2^.

**Figure 3:**
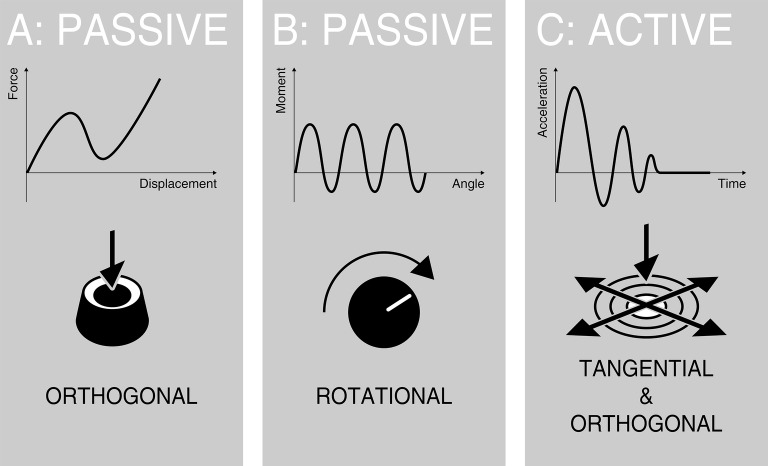
Characteristic haptic curves of a **(A)** passive haptic orthogonal, **(B)** a passive haptic rotational, and **(C)** an active haptic feedback system.

Within confirmation haptics, there is a differentiation between translational and rotational control elements (Bubb et al., 2015). These mainly involve orthogonal movements, such as button presses, or rotary movements (turning knobs). In passive haptic buttons, feedback can be generated by micro-switches, metal domes, and other types of switches in a wide variety of sizes and form factors (e.g., (Alps Alpine Co., 2019; C&K Switches, 2019). By applying orthogonal forces to the surface there is an increase in displacement up to a certain force threshold where a jump in force occurs. This “snap” is felt as haptic feedback. Feedback in rotary knobs is mostly generated by mechanical detents. By turning the knob, the mechanics snap into a detent—producing a click feeling (Reisinger, 2009). The haptic feedback is defined by specifically designed force-displacement-curves (Kühner, 2014; Bubb et al., 2015). Figure 3 depicts characteristic haptic curves of translational and rotational control elements.

#### Active Haptics

*Active haptics* refers to haptic feedback that requires external electrical energy input (MacLean, 2008). Typically, a sensor reacts to tangential (e.g., sliding, swiping) or orthogonal (e.g., press) movements of the user which then triggers an actuator. The actuator moves the interaction surface in a manner often characterized by high acceleration and short travel.

A major advantage of active haptic systems is their programmability and flexibility. Haptic impulses can be changed depending on application and situation. The same technology can be used in different automotive use case such as search and confirmation haptics. In order to design an intuitive interface, we need a clear distinction of various haptic signals in a user setting. Palani and Giudice (2016) argue that empirical parameters for specific active haptic technologies may not be applicable to touchscreen-based haptic perception. Some technologies do not employ pressure-sensitive mechanoreceptors. In addition, other technologies and interaction concepts require active finger movements. However, knowledge on how to implement novel active haptic technologies in an automotive user interface including the typical challenges that need to be considered when applying such technologies are still sparse. This is especially true for impulse parameters. Heijboer et al. (2019) collected subjective descriptions and associations of a variety of piezo-actuated signals and made an attempt to structure these descriptions and associations. Understanding how users experience and describe active haptic signals can aid in successfully implementing active haptics in interface design.

In the field of active haptics, there are numerous applicable haptic technologies. These can roughly be categorized into:

- systems that are moving the interactive surface through the use of an actuator, such as low-fidelity vibrotactile feedback (Klatzky et al., 2014) and high-definition feedback (Lust and Schaare, 2016; Aito, 2018),
- systems that employ friction modulation (Meyer et al., 2014) while sliding over a surface, such as ultrasonic friction (Biet et al., 2007) and electrostatic friction (Bau et al., 2010),
- systems that deform the surface, e.g., electroactive polymers (Matysek et al., 2009) and pin arrays (Culbertson et al., 2018).

For an extensive review of haptic technologies we would like to refer to Banter (2010) and Culbertson et al. (2018).

## A Theoretical Framework of Haptic Processing in Automotive User Interfaces

**Figure 4:**
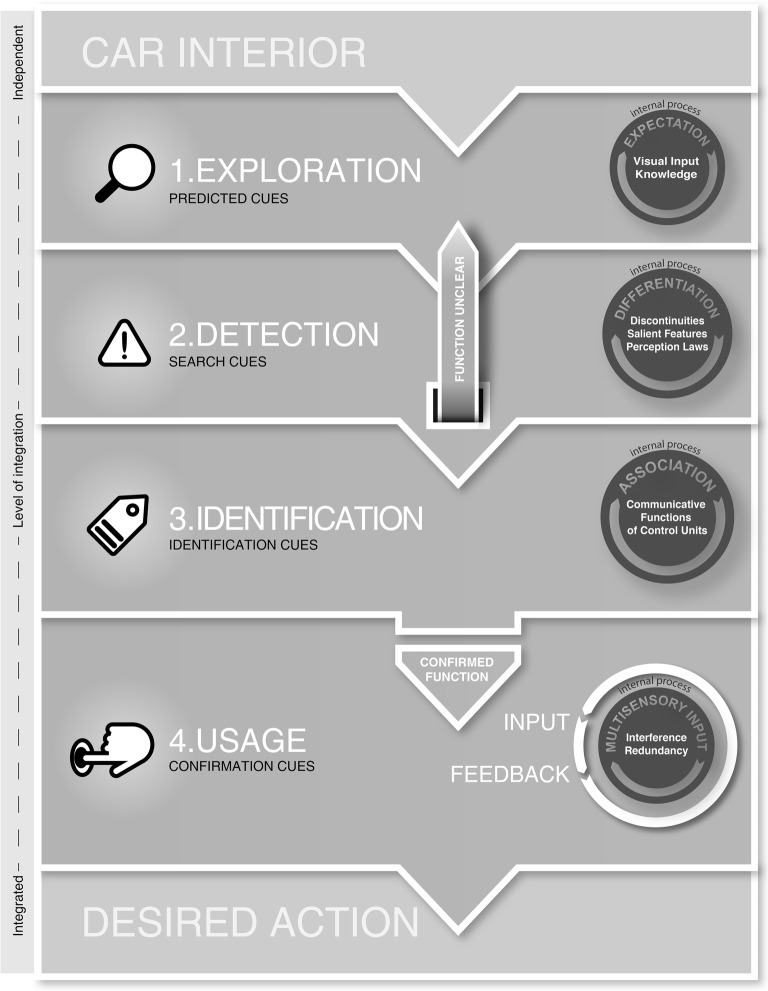
Theoretical framework of haptic processing in automotive user interfaces (fHAPro-AUTO).

The “theoretical framework of haptic processing in automotive user interfaces” (in short fHAPro-AUTO, see Figure 4) focuses on a systematic description of haptic processing in the case of controlling a car’s function. More precisely, it aims to explain in a model-based way, how users perceive and integrate different haptic stimuli during an intentional physical interaction. By constructing the model into discrete phases, crucial steps in the perception that allow for the derivation of guidelines in the design process are shown. In a later section of this paper, there is an outline of a possible study design to validate the proposed phases. The framework is based on existing literature in perception sciences, user interface design and user experience as well as observations from everyday practice. Thus, the model does *not* yet provide empirical evidence but systematizes and harmonizes the given literature.

### Current Discussions on Automotive User Experience

In recent years, haptics within user experiences and within the automotive context has seen growing interest, leading to interesting discussions relevant to the scope of this framework. This section sums up the most important and relevant models and discussions. The general structure of the fHAPro-AUTO is based on other models in UX. The main difference of the fHAPro-AUTO to other discussions and models in UX is the focus of haptic processing in controlling a car’s function including automotive-specific challenges regarding safe and easy-to-use interfaces.

A model in haptic processing not only including perceptual but also cognitive aspects is the “model for haptic aesthetic processing” of Carbon and Jakesch (2013). The general structure of their model and the proposed framework is similar due to the serial nature of haptic processing. Additionally, phase specific top-down processes, such as context of exploration and expectations, and an increasing integration of tactile information and affective evaluations into a holistic mental representation of an interface are aspects that are relevant in both models. A major difference is the focus of the model for haptic aesthetic processing on haptic objects in general, whereas the fHAPro-AUTO is focusing on automotive interfaces.

Gaffary and Lécuyer (2018) provide an extensive review on how haptic feedback can aid various automotive use cases, such as warning and navigation. A classification of how and where haptic information can be conveyed in a car is also given, thus summarizing relevant studies and technologies. In contrast, the proposed framework focuses on how haptic information can be used in the context of controlling a car’s function to support the interaction process. This is mentioned only briefly in Gaffary and Lécuyer (2018).

MacLean (2008) carried out another extensive introduction to haptic interface design, providing valuable information on human perception capabilities and constraints, multimodal interaction, haptic feedback systems, and possible uses of haptic feedback in interaction. However, the focus is on everyday objects, such as mobile phones. Although the proposed framework could also be applied to everyday object interaction, the main intention is to give guidelines for automotive interface design.

An interesting discussion on current haptic challenges with regard to structural and technical aspects was led by Schneider et al. (2017). Here, information on the workflow in the haptic design process was collected from practitioners in different professional areas. They structured common activities, challenges, and also recommended solutions. A strong point is made for the use of psychological research in haptic design by arguing that haptics *“does not end at the actuator”*. Schneider et al. (2017) give general guidelines on how to design with haptic technologies, which is one of the main differences to the automotive scope of the proposed framework. Some of their sub-themes are also relevant in this case, for example haptic latency to create reliable feedback. They argue that hapticians often deal with user constraints, such as designing *feelable but not seeable* interfaces. The present framework aims to deliver more specific guidelines for this challenge in an automotive context.

### General Structure of the Framework

The “fHAPro-AUTO” consists of four discrete and serially proceeding phases with an increasing integration of independent haptic signals into an integrated part of the user interface. The input of the model consists of a car’s interior with a multitude of different interactive and non-interactive surfaces. Surface-integrated or hidden-till-light interfaces may not always be visible at first glance. The starting point of haptic processing is a user’s intention to control a certain function (such as cruise control, volume or temperature) in a car by manipulating a physical control. The four phases – *Exploration*, *Detection*, *Identification*, and *Confirmation* – are described with respect to relevant findings from haptic perception and cognitive research. As haptic stimuli carry valuable information during the perception process, their design is explicitly discussed. Additionally, phase-specific top-down loops modulating haptic processing are discussed. These top-down loops stand for cognitive processes that influence processing at each phase. They can be seen as reoccurring evaluation cycles that start with the beginning of each phase. These phase-internal processes influence processing by facilitating or hindering evaluation of haptic information.

The framework is based on closed-loop control. Information processing in a specific phase proceeds once required haptic information is adequately assessed by the user. In an interaction situation, it seems reasonable to assume that processing and exploration is proceeding once users categorized haptic as valuable to the interaction process.

The framework focuses on haptic processes in touch interactions that require physical contact with certain surfaces by the use of haptic exploration movements. It does not offer descriptions for interaction processes that are conducted by other sensory modalities to touch, such as gesture or speech. Hence, it is not a holistic explanatory approach to describe multimodal user interactions. However, the model takes into account certain multisensory influences on haptic perception during perception and interaction.

### Different Phases of Perception and Top-Down Loops

#### Context

Context heavily influences perception, already at early phases of perception (Carbon and Jakesch, 2013). Contextual information can be given by a certain situation, task, personal mood, experience, cultural background as well as other information the perceived object is carrying. In a car, the context in somewhat fixed, as the passenger’s task is to drive it. Certain relevant driving functions are anchored in a physical user interface, which requires interaction to manipulate. In haptic perception, context cannot only be created by cognitive factors, but also due to material properties and their haptic exploration strategies (Klatzky et al., 1987; Bergmann Tiest, 2010). Haptic materials may feel differently according to which materials were previously touched. For example, the perceived roughness of materials depends on how rough or smooth previously explored surfaces felt (Kahrimanovic et al., 2009). According to research done by Jakesch et al. (2011), functional and aesthetical evaluation of haptic materials rely on contextual information. A car’s brand or experience with different user interface concepts modulate the processing of different materials depending on whether they fit into existing interaction concepts or meet expectations set by the brand name.

#### Processing Phase 1: Exploration

##### Rationale of the Exploration Phase

Haptic processing and exploration of interior surfaces is initiated by a user’s intention to manipulate a physical control in the interior in order to change a certain function. The input of the first stage is a yet unspecified car interior with different interactive areas. In accordance with other models on haptic perception, the initial processing phase is the low-level perceptual analysis of basic surface properties (Klatzky and Lederman, 1993; Carbon and Jakesch, 2013), using various exploration movements (Klatzky et al., 1987). Carbon and Jakesch (2013) presume three different types of exploration during the first encounter with an unknown object: “orthogonal,” “tangential,” and “measure” exploration. Measure exploration means extraction of haptic object information, such as weight and size, but is less relevant in an automotive use case. In an automotive user interaction context, crucial haptic features are mainly extracted by tangential and orthogonal exploration, e.g., sliding or pressing (Götz, 2007). Using these exploration strategies, users do not only extract basic material properties such as roughness, hardness, slipperiness and thermal cues, but also gain knowledge on additional surface features, such as geometries, shapes and textures (Klatzky et al., 1987; Carbon and Jakesch, 2013). Hence, haptic glances (Klatzky and Lederman, 1995) give a “first impression” of interior surfaces. These are first haptic insights on possibly interesting features that are to be further explored, such as bumps or edges. Processing proceeds to the next stage even if there are no special haptic features that invite the user to further exploration, such as on touchscreens. In this case, user might rely on other modalities for further exploration.

This phase sets the starting points for exploration in further phases. The output of this phase is purely physical perception of possibly interesting haptic cues, without including interpretations of possible functionalities. However, perception of basic haptic features is crucial for the integration of different haptic perceptions into a holistic model of the car interior and constitutes starting point for evaluation in following phases.

##### Top-Down Loop 1: Expectations

Haptic perception is influenced by expectations at an early phase (Carbon and Jakesch, 2013). A current, intensively discussed theory in cognitive science is the approach of hierarchical predictive coding (see review by Clark, 2013), which emphasizes the importance of expectations and predefined assumptions in perception. To minimize cognitive load, the human makes assumptions about the external world and only uses sensory input to validate previously formed assumptions. Muth et al. (2016) put predictive coding into an aesthetical context by using predictive coding as an explanation for experiencing pleasure in “decoding” ambiguous artwork. This approach may also be applied in a framework of haptic processing. Perception of early material properties may be used to form hypotheses on possible functionalities of interior surfaces and initiate further exploration. Presumptions based on bottom-up perceptions may function as a priming stimulus. Empirical studies have shown that, for instance, priming can be used to facilitate or hinder perception processes (Palmer, 1975; Albrecht and Carbon, 2014).

Expectations include experience and explicit knowledge on tangible user interfaces concepts as well as prior multisensory, particularly visual, input. Experienced users might skip or fast forward the perceptual process due to their knowledge as to where controls are located and how they are activated. Klatzky et al. (1993) propose a strong influence of prior visual information on haptic exploration in a visual preview model. The user builds up an expectation of previsously viewed objects that are evaluated by touch if the visual input is ambiguous. This is especially important for haptic stimuli as there are tactile properties that can hardly be encoded by vision alone, such as hardness, thermal properties, or slipperiness (Baumgartner et al., 2013). Setting and meeting appropriate expectations becomes even more relevant with the use of seamless user interfaces. This is mainly due to the fact that they lack visual information when interfaces are only shown in specific situations. However, visual prior sets the user’s expectations about how and what user should explore in a control panel. Contrary to today’s daily habits of interaction with touch-sensitive surfaces by tangential swipe and touch gestures, automotive control elements mostly require orthogonal or tangential pressure. If the user’s expectations are not meet, for example, a function is not activated by touching, but pressing or interactive surfaces are not coded with haptic feedback, processing is hindered as the user might be confused. Especially using affordances, a term introduced by Gibson (1979), can help to predict possible user expectations.

At an early stage of haptic processing, it is important to meet the user’s expectations of a user interface by providing appropriate and easy-to-understand haptic information. This is especially important in designing seamless user interfaces.

##### Haptic Information: Prediction Cues

Haptic information processed in this phase are prediction cues. These cues mainly attract attention during the exploration process and invite for further exploration, but are not yet connected to a specific functionality. Users connect them to some kind of relevance, such as separators, orientation and reference points. Therefore, stimulus properties can be widespread to raise attention – textures, gratings, edges, joints, or mere unevenness in a surface. In addition, basic haptic properties, such as hardness, roughness, slipperiness and thermal properties need to be taken into account, as they can shape haptic exploration early on (Carbon and Jakesch, 2013).

#### Processing Phase 2: Detection

##### Rationale of the Detection Phase

The main goal in the detection phase is to detect interactive surfaces and differentiate them from pure design elements. This detection process can be seen as a goal-driven exploration, which is biased by salient haptic features on surfaces. The input of this phase consists of haptic features that were encoded in the exploration phase. Users exploit discontinuities in surface properties but also various exploration strategies to assess whether there is something that can be pressed, pulled, moved or turned. By scanning the surfaces, the user tries to answer implicit questions like “Where is my button?”

In car interiors, separators of interactive and non-interactive surfaces are mostly joints, edges, and recesses. However, not only boundaries but also haptic sensations within an interactive area may be an intuitive and efficient way to indicate interactivity. For example, Lust and Schaare (2016) proposed using unique haptic feedback to code certain content menus on touchscreen-based haptic devices. Surface features such as edges, raised-dots, and even a certain wobbling due to play, may invite direct interaction due to their functional association. Users detect different haptic sensations, such as different materials and textures, and assess their functional purpose. Processing proceeds to the next phase when relevant discontinuities are perceived. The output of this phase is a representation of where interactive elements are on the surface based on perceived discontinuities. Their functionality is yet unknown. However, it is not yet clear, which perceptual input sets interactive areas clearly apart from non-interactive surfaces and which technologies are appropriate to use. Moreover, it is unclear how transitions between different content areas on touchscreen-based interfaces should be coded in terms of haptic feedback patterns to ensure an eye-free operation. To separate different buttons, differentiation *via* a change in perceptual input has to occur.

##### Top-Down Loop 2: Differentiation

Processes influencing perception in this phase ease the differentiation of different haptic stimuli. A basic user interface design principle in graphical user interfaces is to make interactive surfaces and important changes visible (Nielsen, 1994; Harley, 2018). The same is true for tangible user interfaces, where interactive areas should be detectable by distinct haptic features. Visually salient stimuli have been shown to capture perceiver’s attention (Kerzel and Schönhammer, 2013). For an overview of haptic saliency see Kappers and Bergmann Tiest (2015). Due to their pop-out effect in perception, salient features carry valuable context-sensitive information for the user. Carmakers use changes in haptic geometries, edges and joints to separate different buttons, but also to set them apart from mere design surfaces, which are also indicated in Figure 1. This helps the user to blindly find certain controls and keep their eyes on the road. But that is also why a user may effectively be looking for salient stimuli. Users are also guided by prediction cues they perceived in the previous exploration phase. Experimental paradigms, such as haptic search (see also Kappers and Bergmann Tiest, 2015), can help to judge haptic saliency and draw conclusions on the processing of different surface properties. In haptic search paradigms, participants have to decide if certain stimuli properties are amongst the stimulus material they can explore during the experimental procedure. For instance, a high contrast between the target and the distractor stimuli, leads to a fast and easy response action (Lederman and Klatzky, 1997). In user interfaces a high contrast in haptic feedback patterns between control elements and design surfaces facilitates information processing. In contrast, haptic patterns with a low contrast pose confusion and hinder or at least slow down information processing. It seems beneficial to think about specific threshold values that are needed to be reached in order to ensure safe and efficient usage.

Additionally, Gestalt principles (Wagemans et al., 2012a,b) at play when exploring surfaces may influence differentiation. Gallace and Spence (2011b) concluded that most Gestalt laws found for vision are also applicable to haptics. Gestalt psychology shows that humans organize perceptual input in a way that perceived input makes sense. Incorporating different gestalt laws, such as a high figure-ground-contrast, the law of continuation and good Gestalt (Prägnanz) haptic user interface design may be enhanced due to faster processing of different haptic information. For instance, a high figure-ground contrast, meaning using distinct haptic materials for design and interactive surfaces, eases interpretation and processing.

##### Haptic Information: Search Cues

Important haptic features that are encoded in this phase are search cues. Search cues contain salient properties as they are normally used to mark buttons and other control elements. Therefore, they should mainly consist of discontinuities on surfaces. In general, discontinuity means any kind of haptic feedback on in-car surfaces, which means they are technology-dependent. For passive haptics, it is mainly geometries or shapes elevated or indented on a surface (see Figure 1). Examples are edges, raised-lines or raised-dots, detents, joints, recesses or embossments. But also sudden changes of the surface material like a harsh transition from rough to smooth can be interpreted as search haptics. Lederman and Klatzky (1997) found that intensive discrimination features are processed at an early stage, whereas orientation of raised-lines is accessible later on. In active haptics (except for shape-changing technologies), haptic feedback is not physically anchored and constant. Nevertheless, already a perceivable imprecise haptic signal on a flat surface may already feel distinct enough for users. Tunca et al. (2016) tested a seamless button bar with single “click” haptic feedback as separators between buttons. They found slightly higher error rates and distraction compared to a passive haptics counterpart, but see potential with an enhanced haptic design. It is still unclear if different active haptic technologies can be used to generate and simulate classic passive search haptic signals, such as edges or other geometries.

As discussed earlier, such discontinuities may contain relevant information on transitions. In order to design for salient features, perception thresholds (absolute and difference) need to be taken into account. These may vary depending on the technology and hardware setup being used. Palani and Giudice (2016) examined vibrotactile and electro-static parameters for line detection and line tracing. A line with of 1 mm had a 100%-detection rate in both electrostatic and vibrotactile cuing-conditions. Detection rates for thinner lines were better in the vibrotactile condition. Also other usability parameters of touchscreen-based haptic technologies, such as minimum angular magnitude of vibrotactile lines has been researched (Palani et al., 2018). Gershon et al. (2016) compared exploration of an angular stimulus in four different conditions. Angle judgments were good in all conditions; however, exploration was shortest in vision, followed by a tangible (passive haptic) display and a touchscreen device with vibratory and frictional impulses. Properties used for design and interaction surfaces need to be distinctive enough to be perceived as two different materials or impulses. Bergmann Tiest (2010) and Klatzky et al. (2013) give insights on perception thresholds of single passive haptic dimensions, such as roughness and hardness. Bau et al. (2010) examined detection and perception thresholds for electrostatic displays.

In the automotive context, thresholds have to be considered even more conservatively due to physical and cognitive interferences while driving, such as uneven road conditions (Lust and Schaare, 2016). As haptic feedback is often seen as brand-specific, objective values on strength of haptic feedback and activation force are often a matter of disclosure. Therefore, studies are not publicly available.

Beruscha et al. (2017) assume that for easy interaction, users need a reference point on interfaces as already mentioned in Section “Passive Haptics.” This coincides with notions made by Budiu (2019) on the use of virtual buttons on automotive touchscreens. Thus, clearly defined search haptic cues can also be used as an anchor point that provides orientation and enables users to build up a reference frame of the interface. These anchors are starting points for further exploration of interactive surfaces, and the basis of an input movement.

#### Processing Phase 3: Identification

##### Rationale of the Identification Phase

The goal of the identification phase is to clarify the suitable control element for the intended interaction. The input of this phase is the representation of the interface with regard to transitions from interactive and noninteractive elements. Because of the detection phase, users encoded the location of user interfaces. Moreover, they can specify if the interface contains more than just one adjustable element. Yet, the functionalities of single buttons are not clear. In this phase, the user identifies functionality of an interface element, i.e., how the interface can be controlled, but also the precise control element leading to the desired action. Well-designed haptic cues support users with the identification. During haptic exploration within interface boundaries (search cues) perception is enriched with associations on whether the element can be pressed, scrolled, toggled, etc. Additionally, previously perceived search cues are integrated into a holistic representation of the user interface. Both, the unique form factor of buttons on a control panel and spatial information, such as “Is the desired button on the left side of the interface?,” enable the user to differentiate between single elements on a control unit. All this information leads to a confirmed identification, i.e., finding a suitable control element for changing the intended function. Only if the user identifies the suitable control element, haptic processing goes on to the confirmation phase (see Figure 4 “Confirmed Function”). If control elements have been identified falsely, processing is fed into a reoccurring exploration (see Figure 4 Loop “Function unclear”). With ongoing haptic processing, local aspects of user interfaces, such as discontinuities and other surface features, are integrated to generate a holistic perception of in-car user interfaces. The user assigns different meanings to specific control elements. At the end of this phase the user’s mental model, which so far incorporated transitions and locations of interactive elements, is enriched by functionalities of these interactive areas. Hence, in the following phase an input action in the form of pressing or sliding can be performed.

##### Top-Down Loop 3: Association

Cognitive aspects influencing haptic perception in the identification phase are mainly processes that ease an association of haptic with semantic information. Carbon and Jakesch (2013) pointed out that haptic exploration of objects is increasingly enriched by associations as well as aesthetical and functional evaluations. Götz (2007) examined communicative functions of control elements. He found an association between certain design features of control elements (e.g., shape, curvature and fluting of the surface) and perceived functionality. Norman (2013) interprets these “signifiers” as an essential part of interaction design for products to be self-explaining. That means shapes, such as those found by Götz (2007), may trigger semantic associations and previous knowledge as they fit and support specific haptic exploration strategies, such as pressing or grabbing. Using shapes rich in affordances might ease memory retrieval on interaction possibilities. Götz (2007) focused solely on the visual appearance of mechatronic control elements as this is the “first encounter” with an interface. Moreover, he focused on manual controls. However, with technological innovations in interface technologies shapes, textures and other interaction movements, such as swiping, can be used in interfaces. Breitschaft et al. (2019) examined how different haptic shapes can be used to indicate interaction possibilites in automotive user interfaces. They found that participants do implicitly assign functional properties, such as confirmation, more-or-less and selection, to certain shapes. For example, participants associated a horizontal raised-lined with a more-or-less and a solid raised circle with a confirmation functionality. Using affordances posed by different shapes can help user to acquire and operate control elements while keeping their eyes on the road.

User interfaces are easier to understand if control elements consist of haptic information yielding clear associations about functionality. Therefore, using material properties that carry implicit associations about their function may enhance perception and increase user experience.

##### Haptic Information: Identification Cues

Haptic information processing in the identification phase refers to identification cues that goes beyond a mere detection and separation. This means that the user can derive information such as function, differentiation, and movement from how user interfaces are constructed. Götz (2007) focused on design features for an on/off, more-or-less and cursor function. Examples for unique design features are:

- On/off: a convex or concave crown of a surface or elevated circular elements with a revolving chamfer or radius
- More-or-less: circular protruded shape with vertically fluted sides
- Cursor: a spherical segment which is centered to a surface, similar to a trackball on notebook keyboards

These findings are valid for passive haptic manual control elements. Also, the reference surface of a control element contains affordances on interaction. Moving components, such as by pressing or shifting require joints, which in return give feedback which kind of interaction is required. Novel technologies enable new seamless interfaces with new interaction movements, such as swiping. On/off, more-or-less and selection seem to be functional qualities also true for surface-integrated haptic shapes (Breitschaft et al., 2019). It is still a matter of further research, which physical properties are “signifying” design features (shapes and textures) on seamless surfaces. It is also unclear how these findings can be translated to active haptics in order to use them for identification of “virtual” control elements on touch-sensitive surfaces. Surface properties that have previously been used as search cues may also be utilized as identification cues. In the detection phase, stimuli, such as raised-lines, edges and recesses are interpreted as discontinuities, indicating the borders of interactive surfaces. Future studies should focus on the use of active haptic feedback to elicit an interaction movement.

#### External Loop: Function Unclear

The end of the identification phase is a crucial point for the interaction and perception process. The user uses prediction, search and identification cues to fully identify the control element that can be used to change the initially intended function. If the intended control element is found, the interaction process proceeds to the usage phase. If the respective element is not found or the function is still unclear, the haptic processing in the interaction process starts again with exploration, detection, and identification. Insights that are generated by an ongoing haptic search are integrated into subsequent haptic perception processes (see top-down loop *Expectations*). In poorly designed interfaces, where haptic information fails to distinguish between different phases, the user may increasingly rely on information from other modalities for interaction. For example, if the user cannot figure out how a switch is operated by exploring the shape alone, he may take a quick visual look to ease operation.

The fHAPro-AUTO aims to look at haptic cues from a functional point of view. Nevertheless, Sonneveld and Schifferstein (2009) conclude that it is crucial for the product design process to know why users interact with an object the way they do. Besides functional haptic cues, users might further interact with a surface because they find joy in just playing around and experiencing haptic sensations (Carbon and Jakesch, 2013). Tactile materials, such as textiles, natural wood or aluminum provoke arousal and emotions that may be experienced as pleasing, or comforting by users, leading them to a non-functional haptic interaction. Some surfaces may also just look inviting to touch, even though haptic cues are not relevant in the interaction process (Klatzky and Peck, 2012). Therefore, the act of touching may also only be initiated for hedonic reasons.

#### Processing Phase 4: Usage

##### Rationale of the Usage Phase

In the usage phase, the user manipulates the previously identified function using haptic feedback. Input from the previous phase includes a representation of where and how elements within the interface can be manipulated. Haptic cues that are perceived during direct interaction are directly connected to a change of the operation status of the function. These cues are interpreted as a confirmation of input actions, which can be switching a function on/off, adjusting more-or-less or selecting items by using a cursor or scrolling (Götz, 2007). Especially for continuously adjustable control elements, an ongoing evaluation of input and feedback takes place. By turning, sliding or pushing the user tries to collect precise information about the relation of confirmation cues and their representation in the user interface. Ongoing confirmation cues are evaluated with respect to the targeted action. That means the user is further performing the input movement indicated by the control element, until the targeted output is reached. Confirmation cues are used to verify that the user is reaching, or has reached, the intended goal. Once the desired target is reached the interaction is completed.

##### Top-Down Loop 4: Multisensory Input

Processing of haptic information in the usage phase is modulated by multisensory input. Human perception and judgments in complex situations do not rely on a single modality. In-car user interaction is increasingly enriched by multimodal input and output technologies. According to the “modality-appropriateness” hypothesis by Welch and Warren (1980), the modality that is most suitable for encoding specific features of an object is dominating the perception and evaluation process. As seen in an earlier Section “Top-Down Loop 1: Expectations,” multimodal feedback can be very useful in in-car interactions. The user may rely on auditory cues, such as an increase in song volume, as confirmation. In this case, haptic information may be neglected, because auditory information is a more reliable source. Modality-appropriateness is also discussed with regard to theories on multisensory integration (Ernst and Bülthoff, 2004). This is likely to be true if additional visual feedback, for example a graphical user interface on a screen is provided. According to Stein and Rowland (2011), multisensory integration of cross-modal stimuli follows three basic principles: spatial, temporal and inverse effectiveness. This means integration is more likely when cross-modal stimuli are presented at the same time or place. In this context, inverse effectiveness means that the strength of an individual unimodal stimulus is inversely related to the strength of multisensory integration. A relatively weak response to stimuli presented in a unimodal setting can also enhance integration. Therefore the implementation of multimodal feedback has to be considered carefully, especially with regard to interferences between sensory inputs from different modalities. Car manufacturers incorporate additional acoustic feedback into haptic user interfaces. Tikka and Laitinen (2006) found the biasing effect of audio feedback on perceived haptic strength in piezo actuated devices to be weaker than anticipated. Haptic intensity evaluation was biased more with higher sound levels than with lower sound levels. Presenting redundant information on more than one perceptual channel enhances user experience in interaction situations (Pitts et al., 2009, 2012b). Reddy et al. (2009) showed that multisensory redundancy increased speed and accuracy in user interfaces for elderly users. Multisensory information may ease and speed up processing due to processes such as familiarity and mere-exposure (Zajonc, 1968; Jakesch and Carbon, 2012).

MacLean (2008) concludes that for multisensory interfaces, enhancement as well as inhibition effects need to be discussed. Depending on its quality, subsequent stimuli can inhibit or facilitate a prior perceived one. But also primary stimuli can influence the perception of subsequent input, which is called sensory adaptation. For example, Kahrimanovic et al. (2009) found that textures felt after a rough stimulus felt smoother compared to textures felt after smooth stimuli. Breitschaft and Carbon (2016) found that adaptation effects may also be evoked in visuo-haptic settings.

##### Haptic Information: Confirmation Cues

Crucial haptic information in the usage phase are confirmation cues. The information is directly linked to the operation of the control element. In translational control elements, such as buttons or rocker switches, the transition from one state to another is characterized by a button snap. Input gestures on touch-sensitive surfaces, such as sliding, can also be supported by confirmation cues, for example by using active haptic systems. In rotational control elements, detents felt by turning usually indicate the change of an increment in the respective function. Button snaps or detents need to trigger a clear and distinct haptic feedback. A weak feeling might lead to ambiguity as to whether or not the control has been operated. Haptic feedback in passive translational or rotational control elements can be precisely determined with force-displacement-curves (see Figure 3). Numerous studies have researched the connection between physical parameters and subjective assessments to give an optimal feedback in translational (Reisinger, 2009) and rotational control elements (Kühner, 2014) for confirmation haptics. Specific force-displacement-characteristics can be used to evoke specific emotions (Rösler et al., 2009) to create a certain user experience in car interiors (for example, sporty vs. luxury). Furthermore, customers are able to differentiate between different button-feelings (Wellings et al., 2008). Wellings et al. (2010) found that participants used factors like “image,” “build quality,” and “clickiness” to characterize and differentiate between multiple buttons.

There is some research on the subjective evaluation of active haptic feedback technologies. Koskinen et al. (2008) found that a tactile click for a virtual button on a mobile device produced by a piezo actuator felt slightly more pleasant than a vibration motor. Salminen et al. (2011) studied affective evaluations of piezo actuated haptic and auditory feedback. Haptic stimuli with a longer rise time were found to be pleasant. Most of these studies are done in the context of mobile devices, but implementation of active haptics systems in car interiors is different, which is why generalizability to an automotive context is limited (Schneider et al., 2017).

Additionally, confirmation cues need to be easily separable from search and identification cues, in order to decrease ambiguity of haptic cues. Haptic cues can become more ambiguous if search and confirmation cues are generated by the same actuator, for example in active haptic systems. This lack of precise differentiation, for example between the feeling of a button area and the feeling of the button snap, may lead to dissatisfaction. Passive haptic feedback solutions are less susceptible to this problem, as search and confirmation cues already yield a fairly different haptic sensation. Passive search cues, such as textures and shapes, are clearly separated from confirmation cues, such as a button snap. This analogy could be applied to active haptic systems, which mean haptic feedback in both cases should yield a clear and distinctive haptic experience.

#### Desired Action

The output of the framework is an integrated mental model of haptic feedback impulses in the car. This leads to the previously desired action. After an ongoing evaluation of different haptic stimuli during interaction, the user not only reached the appropriate control element but also changed it to the required operation status. The interaction process has ended. Insights gained from the complete interaction process are integrated into previous cognitive models, further expectations, and knowledge that are exploited in future interaction situations (see “Level of Integration” in Figure 4).

#### Affective Evaluation

Aesthetic evaluation is an important part of user experience design. At the end of the interaction process, the user has integrated haptic information from single phases into a holistic mental model of the interface. In addition to functional evaluation of the haptic information, the user is evaluating haptic information based on personal preferences (Carbon and Jakesch, 2013). These insights are integrated into the previously formed mental model of the interface. It was already mentioned that especially material properties, for example, wood, leather, metal, or plastic, have an impact on pleasantness and liking (Gallace and Spence, 2011a). Also latency and timing seem to be an important factor, influencing perceived quality of an interface (Kaaresoja et al., 2014). These affective evaluations play a role in reoccurring interaction processes as they influence expectations.

## Application of a Framework of Haptic Processing in User Interfaces

**Figure 5:**
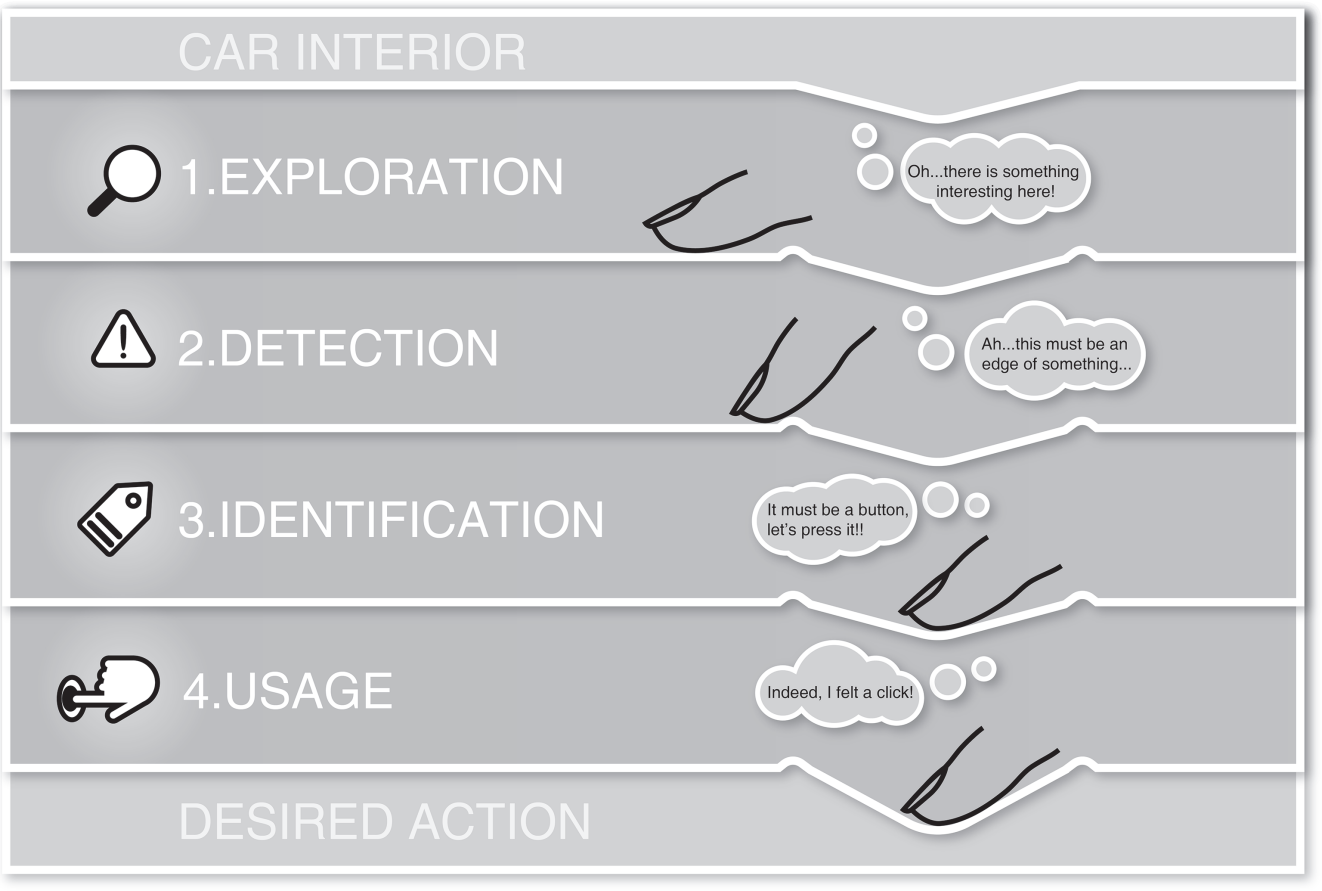
Application of the framework of haptic processing in automotive user interaction in an exemplified interaction situation. This figure shows the four different phases of the framework with the respective actions performed by the user.

The above-propagated framework describes the perception process of haptic information involved in controlling a car’s function (Figure 5). It gives insights on perceptual and cognitive processes during haptic interaction, which is a rather theoretical approach. However, one aim of this model is to provide guidelines on how to optimally utilize haptic information during the design process of automotive user interfaces by including already existing literature. Basic guidelines can already be drawn (see Table 2) from the framework. Further studies are required to deduct more precise guidelines at single phases of processing. In this section, we would like to show how this framework can already facilitate the design process of tangible user interfaces in cars. Looking at the different user interfaces in Figure 1 demonstrates which guidelines aid the effective design of haptic feedback in automotive interfaces. Passive haptic control panels in recent cars often already follow these design principles (see Figures 1A,B). For example, visual and haptic feedback information are present, as are edges and joints. Additionally, confirmation feedback, mostly through button pressing or knob turning is vastly different from search haptic feedback. Particularly in seamless touch-sensitive and touchscreen-based surfaces, the following guidelines may be useful (see Figure 1C), as such interfaces often lack haptic feedback.

**Table 2:** Connection of phases of the framework and a first set of derived guidelines.

Phase	Guideline	Description
Exploration	Holistic user experience	Make use of the haptic experience that is inherently connected with certain technologies
Detection	Clarity	Make transitions between different semantic areas feelable
Identification	Intuitiveness	Make use of affordances posed by certain technologies to indicate interactivity
Usage	Discriminability	Make search and confirmation cues distinct from each other to avoid ambiguity

### Holistic User Experience

In an automotive context haptic feedback, first and foremost, needs to ensure safety of use, reduce cognitive load and distraction. Usability research mostly covers objective and measurable aspects of haptic feedback. As shown in Pitts et al. (2012b) perceived strength of haptic impulses are of great importance for a positive UX. The proposed framework helps to understand how haptic feedback can be successfully integrated into tangible user interfaces by dividing the perception process into *Exploration*, *Detection*, *Identification*, and *Usage*. Haptic feedback that guides and supports interaction enhances user experience.

Several studies show that incorporating haptic feedback into user interfaces also increases acceptance among users (Pitts et al., 2009, 2010; Weddle and Yu, 2013) and adds value to a product. Active haptics allows more aesthetically pleasing interfaces compared to passive haptic counterparts (Tunca et al., 2016). Designers also need to take aesthetical evaluations of haptic feedback into account (haptic aesthetics). Switches not only need to work, but also need to feel high quality in order to yield pleasure and fascination (Carbon and Jakesch, 2013).

One aspect contributing to a high quality UX is latency, which is especially crucial in haptics and even more in the automotive context. Schneider et al. (2017) emphasized the importance of latency and timing. Not only does haptic feedback need to be delivered fast, but also needs to be synchronized with other feedback modalities, such as a confirmation sound and a haptic click. Kaaresoja et al. (2014) found that in the tactile domain, the point of subjective simultaneity of two stimuli is 5 ms. Furthermore, the perceived quality significantly drops if latency is higher than 70 ms from interaction to feedback. They suggest a latency of 5–50 ms for tactile, 20–70 ms for audio and 30–85 ms for visual feedback. These values are in line with the conclusion drawn by Weddle et al. (2013) who performed an automotive-specific study. Jay and Hubbold (2005) point out that users’ seem to tolerate higher haptic feedback latency if an easy task is at hand.

Haptic feedback has the potential to create unique and brand-specific design features. One of the earliest and most widely used examples in the automotive industry is the *iDrive* controller by BMW (Bernstein et al., 2008). Using touchscreens and touch-sensitive surfaces limit these idiosyncratic, brand-specific design features (“Formensprache” as called by Carbon, 2010).

### Clarity

In recent years, there has been a staggering increase in touchscreens and touch-sensitive surfaces in cars. Design surfaces become interactive without any visible borders between control panels. Especially with a hidden-till-light effect, user interfaces are at times invisible (Aito, 2018). This impairs blind control and forces users to look where their finger is placed. However, in control panels buttons should at least remain perceptible through touch. For example, using piezo-actuated systems, the edges of buttons could be indicated by a simple “click” sensation that is triggered when crossing a virtual boarder. Nevertheless, an interactive area could also be represented by a texture that is felt by sweeping across a surface. It is not yet clear which haptic information is most suitable to enable users an easy detection of control elements on seamless surfaces.

Parameters have to be set in a way that impulses are always perceivable to avoid confusion. For active haptic stimuli, this means not only the strength but also waveform and temporal aspects, such as line width (Palani and Giudice, 2016), of an impulse. In in-car applications, especially when using vibrotactile stimuli, external factors, such as vehicle vibration, need to be taken into account when setting impulse parameters. Thus, perception thresholds reported in lab settings are a mere starting point in interface design. Beruscha et al. (2016) are currently working toward user requirements for touch screen interactions.

### Intuitiveness

Haptic feedback in automotive user interfaces can support intuitiveness of user interfaces. Especially with touch-sensitive interfaces, there is an increasing number of input possibilities than mere pressing. This may be confusing for users, because they do not understand how to interact with a plane interface. Employing signifying design features, such as found by Breitschaft et al. (2019), Götz (2007) and Mueller (2016) for passive haptic stimuli, ease interaction. Designers should make of user’s association and affordances of shapes and impulses during haptic perception to increase ease-of-use. For example, an on/off function should be manifested in a recess or ridge. If users scan across a surface, almost falling into a recess with the finger is associated with pressing.

### Discriminability

In previous sections, we described that haptic processing in user interfaces is a staged process with crucial phases. In order not to confuse users, haptic stimuli should be designed to distinguishably meet requirements set in each phase. More precisely, search cues need to feel differently than confirmation cues. For passive haptic interfaces, discriminability is relatively easy to obtain as search haptics yields a fairly different haptic experience as confirmation haptics. If only one active haptic actuator solution is used in the interface, discriminability becomes an issue. Even though development kits of various tech suppliers offer a wide variety of adjustable parameters, haptic impulses may feel rather similar. Similar to passive haptic control elements, designers should aim for eliciting a distinct haptic experience. For example, in technologies involving vibration, waveforms of impulses could be set in a way that the confirmation feels like pressing a real button, whereas search haptics could feel like a sharper impulse, as if one would go over an edge.

### Future Work

This paper summarized findings from research in the field of haptic feedback, with the aim of generating guidelines to aid in the design of automotive interfaces. It also identified areas with open research questions. For example, it remains unclear which parameters are best suited to differentiate between search and confirmation cues. The framework could also be used as a model of haptic information processing. However, empirical data on the validity of various phases, and the model itself, are not yet available. Future research aims to check the sequential structure of the model and the importance of single phases. A target-select-and-confirm paradigm could include a number of different haptic feedback scenarios, involving both search and confirmation haptics. Video recording or a think-aloud approach could yield insights into how users explore surfaces and how they utilize haptic information. An example of concrete hardware to be used in the study could be a touchscreen or a touchpad with haptic feedback, programmed to interact with a graphical user interface. Various items could be coded with different haptic feedback patterns. The goal of the study would be to determine the optimal haptic representation of certain elements for a variety of use cases involving search, identification and confirmation feedback.

Future research could also involve gathering feedback on the proposed framework from experts, both within the automotive industry, as well as other industries that deal with the design of seamless interfaces.

## Conclusion

Studies on automotive haptic feedback mostly focus on increasing driving safety and blind operation in control elements. They show that haptic feedback can positively enrich in-car user experience. However, the scope of these studies is mostly to validate a certain technological solution. Little research deals with the optimal design of haptic features and how haptic feedback can support the user in searching for control elements. The theoretical framework presented in this paper describes the process of haptic perception and points to crucial phases in perception that need to be addressed by interaction and technological concepts. It also gives rough guidelines as to how distinctively designed haptic features can support the interaction process and enhance user experience. This gets even more important with the surface integration of control elements, where functional and aesthetical aspects of haptic perception are even more connected. Psychologically motivated models on haptic perception, such as the one proposed in this paper, may support practitioners such as designers and engineers as well as empirical researchers in understanding the shortcomings and capabilities of human haptic perception in automotive user interfaces.

## Author Contributions

SB had the initial idea to harmonize findings in automotive haptic feedback in a common framework and terminology. C-CC brought insights from haptic aesthetic processing and perception research in automotive contexts. SB did the main part of the research, mainly wrote the manuscript and worked on rough drawings of figures. SB, SC, and C-CC worked further on the manuscript.

### Conflict of Interest Statement

The authors declare that the research was conducted in the absence of any commercial or financial relationships that could be construed as a potential conflict of interest.

At the time of researching and writing this paper SB was enrolled in the PhD-programs at BMW Group and Bamberg Graduate School of Affective and Cognitive Sciences (BaGrACS), supervised by C-CC. SC was employed by BMW Group. C-CC. declares no competing interests.

## Footnotes

^1^The expression User Experience (UX) was first broadly used by Norman (2013). UX comprises all of a user’s experiences with a product. This not only entails the user’s affective and cognitive associations but also expectations. The focus of user experience design is to meet user’s requirements in interacting with a product.

^2^The bumps on the *F* and *J* keys on a keyboard are an easy, but effective way of how haptics can aid in everyday-life. The bumps provide easy finger orientation and reference frame for 10-finger typing—some new notebook keyboard layouts do not provide them anymore, unfortunately, and: users indeed lose control in a quite implicit way.
